# Epidemiology of gall bladder cancer and its prevalence worldwide: a meta-analysis

**DOI:** 10.1186/s13023-025-03652-0

**Published:** 2025-03-27

**Authors:** Ali Afzal, Yan-Yan Liu, Amara Noureen, Amna Rehman, Mehreen Iftikhar, Hanan Afzal, Fareeha Azam, Umair Ali Khan Saddozai, Tayyba Jan, Zoya Asif, Lei Zhang, Xin-Ying Ji, Muhammad Babar Khawar

**Affiliations:** 1 Institute of Translational Medicine, Medical College, Yangzhou University, Yangzhou, Jiangsu China; 2 Molecular Medicine and Cancer Therapeutics Lab, Department of Zoology, Faculty of Science and Technology, University of Central Punjab, Lahore, Pakistan; 3 Medical College of Xuchang University, Xufan Rd, Weidu District, Xuchang City, 461000 Henan China; 4 Applied Molecular Biology and Biomedicine Lab, Department of Zoology, University of Narowal, Narowal, Pakistan; 5 Institute of Zoology, University of the Punjab, Lahore, Pakistan; 6 Department of Zoology, Lahore College for Women University (LCWU), Lahore, Pakistan; 7 Henan International Joint Laboratory for Nuclear Protein Regulation, School of Basic Medical Sciences, Henan University, Kaifeng, Henan China; 8 Center for Molecular Medicine, Faculty of Basic Medical Subjects, Shu-Qing Medical College of Zhengzhou, Gong-Ming Rd, Mazhai Town, Erqi District, Zhengzhou, 450064 Henan China; 9 Department of Nuclear Medicine, Henan International Joint Laboratory for Nuclear Protein Regulation, The First Affiliated Hospital, Henan University College of Medicine, Ximen St, Kaifeng, 475004 Henan China

**Keywords:** Gallbladder cancer, Prevalence, Epidemiology, Risk factors, Global trends, Cancer research

## Abstract

**Background:**

Gallbladder carcinoma (GBC) accounts for 1.3% of cancer incidence and 1.7% of cancer-related deaths which emphasizes the need for comprehensive research in epidemiological trends.

**Aim:**

We aim to address this gap by investigating global prevalence trends across various regions, age groups, risk factors and cancer stages.

**Methods:**

A meta-analysis of studies retrieved from Google Scholar, PubMed and Web of Science, reporting prevalence of GBC was conducted using a predetermined screening criterion. Meta Regression and Egger’s Regression-based tests were employed to assess heterogeneity and publication bias, respectively.

**Results:**

We identified three types of studies (*n* = 20), primarily originating from Asia (*n* = 10) over a cumulative time period of 24 years (1988–2012). The pooled analysis revealed a statistically significant GBC prevalence of 20.3 ± 5.2% (95% CI 9.3–31.3%, *p* = 0.001) among at-risk populations, including those with gallstones or cholecystitis. Analysis of potential publication bias showed none, nevertheless, individual parameters indicated varying significance. Subgroup analyses highlighted regional, temporal, and demographic variations, emphasizing the influence of factors like sample size and age on GBC prevalence. Correlation analysis demonstrated strong positive associations with sample size (*p* < 0.01), gender distribution (male: r = 0.85, *p* < 0.01, female: r = 0.806, *p* < 0.01), and prevalence rates (r = 0.98, *p* = 0.04).

**Conclusion:**

Despite of less data present, our comprehensive overview of prevalence, regional variations, and demographic associations serves as a crucial starting point for future targeted investigations. The study fulfills a gap in epidemiology of GBC and emphasizes the need for increased attention and provides a pioneering arena in future.

**Supplementary Information:**

The online version contains supplementary material available at 10.1186/s13023-025-03652-0.

## Highlights

- Gallbladder carcinoma (GBC) accounts for 1.3% of cancer incidence.
- Our meta-analysis estimates the prevalence of GBC at 20 ± 5% (*p* = 0.001).
- Topography, study duration, design, age groups and sample size revealed significant variations.
- Larger sample potentially influence the prevalence (*p* = 0.040).
- Positive associations (*p* < 0.01) between prevalence and sample size, males and females were showed.

**Supplementary Information:**

The online version contains supplementary material available at 10.1186/s13023-025-03652-0.

## Introduction

Gallbladder carcinoma (GBC) is relatively rare tumor in clinical studies but it is one of most common cancer of biliary tract. Detection of GBC cancer at early stage is challenging owing to its poor diagnosis method for detection of this cancer type along with its rare symptomatic representation [1]. Consequently, GBC is often discovered incidentally during cholecystectomy procedures conducted for gallbladder stone indications. The lack of effective diagnostic tools leads to the identification of the cancer at advanced stages which impedes proper treatment due to poor prognosis and limited therapeutic alternatives.

Worldwide GBC related deaths accounts 0.9% and 0.6% of newly diagnosed cancers. In 2020, globally 10 million cancer-related deaths reported out of 19.3 million cancer diagnoses [2]. Further, GBC accounts for about 1.3% of total cancer incidence and 1.7% of total cancer related deaths worldwide [3]. The incidence of GBC have been investigating globally in many developed countries including South Korea, United States, Canada, United states, United Kingdom, New Zealand and Australia [4]. This underscores the need for comprehensive research efforts that can bridge the existing knowledge gap and provide a more nuanced understanding of the global landscape of GBC prevalence.

A few decades later, GBC was poorly understood with complex association with lifestyle, genetics, metabolic factors and other factors related to development of carcinogenesis [5]. Therefore, given its distinctive geographical distribution, asymptomatic presentation, and low survival rates [6], there is a pressing need for increased research efforts to address the existing gap in understanding all aspects related to GBC. Furthermore, as far as we are aware, there has not been any study examining the prevalence of GBC across diverse parameters. Closing the knowledge gap through additional validation studies on GBC prevalence is crucial for enhancing our understanding to promote early detection, and improve outcomes for those affected by this challenging cancer.

Herein, we aim to investigate the worldwide prevalence trends of GBC to fill the important gap in the literature. Moreover, our study provides a comprehensive overview of prevalence rate across various geographical distributed regions, age and cancer stage.

## Methods

### Search strategy

The current study was conducted in accordance with the published guidelines of conducting non-randomized studies [7]. Authors independently conducted search on PubMed, Google Scholar, Scopus and Web of Science using relevant keywords (Supplementary Material: Search Strategy) and records till November, 2023 were accumulated in a reference managing software (EndNote v21.2, Bld: 17,387) for further processing and screening. No time frame or language restrictions were applied due to rarity of studies.

### Screening and data extraction

Two authors independently reviewed the records to exclude the type of published literature (reviews, conference abstracts, letters to Editor and correspondences), study design (screening studies, ecological data-based studies and case–control studies), missing data on prevalence, reports on incidence or mortality and risk assessment studies. Only original research comprising; cross-sectional, descriptive studies, population-based cancer registries and retrospective studies reporting the demographic details (mean age, age distribution, and gender distribution etc.) and clinicopathological features (such as; cancer staging and associated risk factors) with prevalence of GBC were included.

Full-text articles of included records were obtained and data variables were extracted from individual studies by two authors independently. The extracted data include; authors, year of publication, study title, country, study duration, study design, sample size, affected individuals, mean age, number of males and females, male/female ratio, associated risk factors, cancer stage, and prevalence.

### Quality appraisal and data analysis

Included studies were assessed as per guidelines of Joanna Briggs Institute for prevalence studies (Supplementary Material: Quality Appraisal) and categorized on the basis of various categories, e.g., geographical region, study duration, study design, sample size, mean age, cancer stage and risk factors. The analysis was conducted using IBM SPSS Statistics v28.0.1.1 under subscription-based license, as per published guidelines by Sedat et al., (2022) [8], using binary outcomes with pre-calculated study outcome via Freeman-Tukey double arcsine transformation [9] while standard errors and variances via the given formulae;

$$ SE\left( P \right) = \sqrt {\mathop \sum \limits_{i} {\raise0.7ex\hbox{$1$} \!\mathord{\left/ {\vphantom {1 {Var\left( {p_{i} } \right)}}}\right.\kern-0pt} \!\lower0.7ex\hbox{${Var\left( {p_{i} } \right)}$}}} \;and\;Var\left( p \right) = \frac{{p\left( {1 - p} \right)}}{N} $$

respectively as described in a study by Barendregt et al. [10]. Restricted maximum likelihood (REML) was used to estimate unbiased-between-study variance [11] while truncated Knapp-Hartung adjustment was used for standard error adjustment [12]. While the male and female sample sizes are not statistically independent, appropriate statistical techniques, including stratified analysis and multivariate adjustments, were employed to account for this dependency. Subgroup analyses based on each category were performed with same protocol. Meta Regression was performed with moderators as a covariate and funnel plot was drawn to assess potential sources of heterogeneity. Publication bias was assessed via Egger’s Regression-based test including intercept regression and statistics estimation based on t-statistics between effect size and standard errors of individual studies. Bubble plot and funnel plots were drawn to further assess the results.

## Results

### Study characteristics and descriptives

**Fig. 1:**
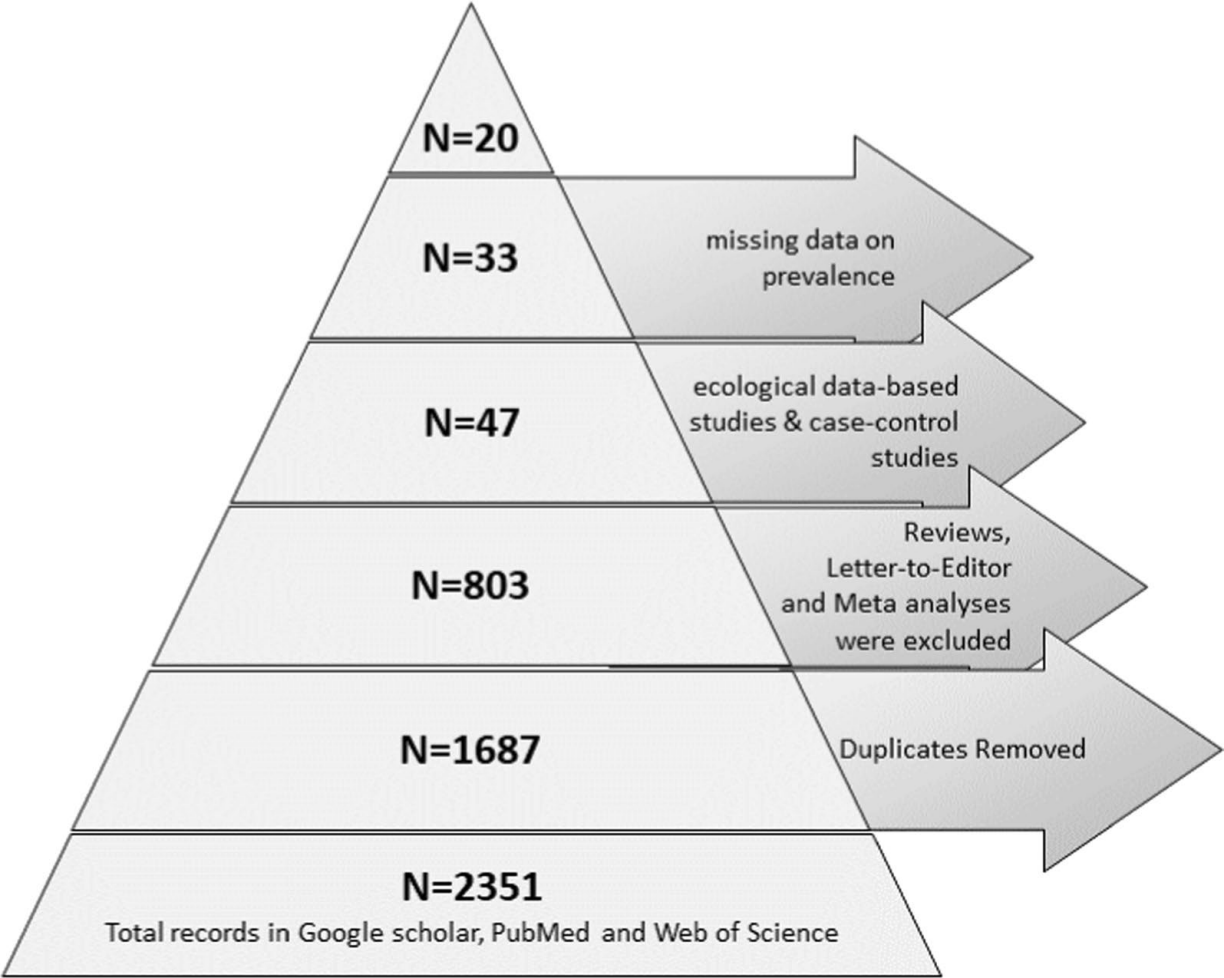
Flowchart illustrating the systematic selection process. The screening process resulted in 20 eligible records after excluding duplicates, other than prevalence-based research and studies with missing details

Among the 2351 records identified across various databases, the screening process led to the inclusion of 20 qualifying studies (as delineated in Fig. 1 and summarized in Table 1). Notably, half of these studies (*n* = 10, 50%) emanated from various Asian countries, while the remaining 10 studies originated from America (*n* = 7, 35%), Africa (*n* = 1, 5%), Australia (*n* = 1, 5%), and Europe (*n* = 1, 5%). Our collection comprised three distinct study types: cross-sectional descriptive studies (*n* = 7, 35%), retrospective studies (*n* = 10, 50%), and studies utilizing population-based cancer registries (*n* = 3, 15%). The time period of the selected studies ranged from a minimum duration of 2 years to a maximum of 24 years, which was conducted from 1988 to 2012. All studies included in our analysis were published between 2003 and 2022. Notably, Kim et al. (2016) reported a prevalence of 6.0% among a population of 115,178 undergoing ultrasonography in Korea over 14 years from 2001 to 2015. This high prevalence is attributed to their focus on high-risk individuals, including those with gallbladder sludge, which is strongly associated with malignancy risk. The descriptive statistics, shown in Table 2, reveal significant variability in prevalence (Mean = 7.78 ± 3.567 SD = 15.95) and sample sizes (Mean = 1.4 × 10^3^ ± 6097.972, SD = 27,270.96) ranging from 200 to 115,178 individuals. However, mean age (Mean = 59.03 ± 2.141, SD = 9.08) reported in studies showed moderate variability. Moreover, the data on mean age showed slightly non-normal distribution in mean age (Skewness = − 0.3 ± 0.54, Kurtosis = − 0.39 ± 1.04).

**Table 1:** Study characteristics of studies reporting the prevalence of Gall Bladder Cancer

Authors	Year	Country	Study period	Sample Size	Mean Age	M/F Ratio	Risk factors	Cancer stage	Prevalence	References
Silva et al	2022	Brazil	2018–2022	642	43.9	2:3	Cholecystitis	N/A	0.16%	[13]
Basak et al	2016	Turkey	2009–2013	1747	48.7	2:3	Cholecystitis Gallstones	I–III	0.23%	[14]
Al Manasra et al	2018	Jordan	2002–2016	11,391	68	4:3	Gallstones Gallbladder polyps	N/A	0.27%	[15]
Fonseca et al	2022	Chile	2016–2019	3270	71	N/A	N/A	N/A	0.3%	[16]
Tekeşin et al	2018	Turkey	2009–2017	3856	54.8	7:6	N/A	II–IV	0.33%	[17]
Martins-Filho et al	2015	Brazil	2007–2010	2018	N/A	N/A	N/A	N/A	0.34%	[18]
Lilic et al	2015	New Zealand	2003–2013	4128	70.6	1:3	Adenocarcinoma	I–IV	0.44%	[19]
Raina et al	2016	India	2014–2016	464	41.1	0:3	N/A	N/A	0.6%	[20]
Malhotra et al	2017	India	1988–2012	12,410	57	3:4	N/A	N/A	0.6%	[21]
Bani-Hani et al	2003	Jordan	1994–2000	4502	61.4	26:7	Cholecystitis Gallstones	I–IV	0.73%	[22]
Olusola-Bello et al	2021	Nigeria	2015—2017	1191	60.3	7:8	Gall Stones	III–IV	1.25%	[23]
Apodaca-Rueda et al	2017	Brazil	2010–2015	893	60.23	3:8	Gall Stones Gallbladder polyps	I–IV	1.3%	[24]
Maheshwari et al	2020	India	2014–2018	31,355	60.5	2:5	N/A	N/A	3.8%	[25]
Lohana et al. -	2009	Pakistan	2006–2008	200	53	3:3	Gall Stones	N/A	4%	[26]
Bertran et al. -	2010	Chile	1998–2002	317	66.36	1:3	Gall Stones	I–IV	4%	[27]
Roa et al.	2014	Chile	1987–2005	29,840	55.83	1:4	Cholesterolosis, Obesity	N/A	4.6%	[28]
Alkhayyat et al	2021	Unites States	1999–2019	4790	73.5	3:5	Gallstones Obesity Diabetes Mellitus	I–III	4.8%	[29]
Faivre et al.	2012	Europe	1995–2002	50,646	N/A	N/A	N/A	I–IV	2.1%	[30]
Singh et al.	2021	India	2009–2014	2610	53.49	7:6	N/A	N/A	4.31%	[31]
Kim et al.	2016	Korea	2001–2015	115,178	62.8	3:2	N/A	N/A	6%	[32]

**Table 2:** Descriptive statistics of key variables

	Mean ± SE	Range	Std. Deviation	Variance	Skewness	Kurtosis
Prevalence (%)	7.78 ± 3.567	0.16–60	15.95	254.41	2.65 ± 0.51	6.57 ± 0.99
Study duration (years)	7.95 ± 1.47	2–24	6.57	43.21	1.27 ± 0.51	0.58 ± 0.99
Sample size (n)	1.4 × 10^3^ ± 6097.972	200–115178	27,270.96	7.44 × 10^8^	3.08 ± 0.51	10.4 ± 0.99
Mean age (year)	59.03 ± 2.141	41.1–73.5	9.08	82.48	− 0.3 ± 0.54	− 0.39 ± 1.04
No. of males	2527.88 ± 2433.843	0–41464	10,034.99	1.01 × 10^8^	4.12 ± 0.55	16.99 ± 1.06
No. of females	1797.41 ± 1617.303	3–27,642.8	6668.31	4.45 × 10^7^	4.11 ± 0.55	16.9 ± 1.06

### Overall analysis

**Fig. 2:**
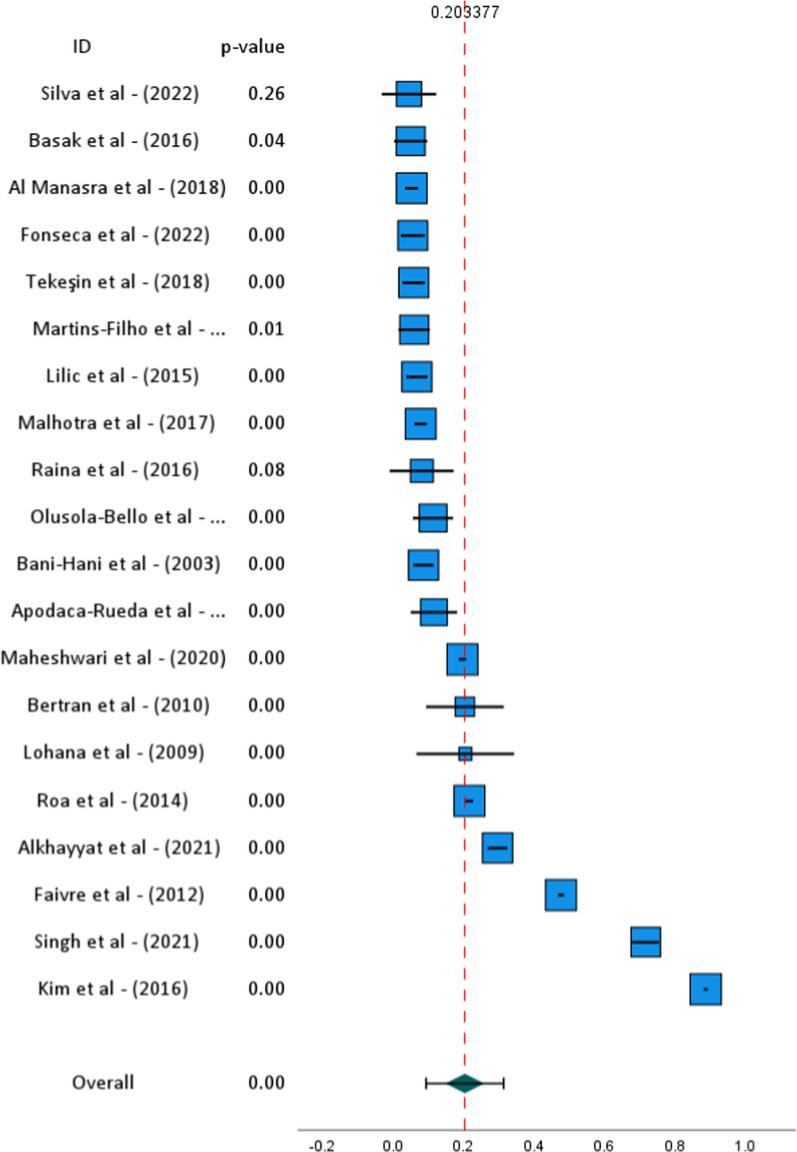
Forest plot highlighting each study with its individual effects. All the studies showed a statistically significant effect in pooled prevalence with overall *p* = 0.00, except Silva et al. [13] and Raina et al. [20] showed non-significant outcomes in the study collection

Overall analysis using random effects model revealed statistically significant prevalence of gallbladder cancer worldwide at 20.3 ± 5.2% (95% CI 9.3–31.3%, *p* = 0.001) across the studied populations, as shown in Fig. 2. Although, the prevalence is not representative of the general population but rather reflects the proportion of GBC cases among populations at risk, such as individuals with gallstones or other predisposing conditions. The narrow confidence interval (95% CI 9.3–31.3%) enhances the reliability of this finding by indicating consistent patterns across studies despite significant heterogeneity (Tau^2^ = 0.05, H^2^ = 630.26, I^2^ = 100%, *p* < 0.001). Potential sources of heterogeneity have been explored in the next section, which likely reflects variations in study design, geographic regions, and sample characteristics. Interestingly, overall pooled effect (z = 3.87, *p* < 0.001) emphasizes a significant association between GBC prevalence and the studied factors.

### Publication bias

**Fig. 3:**
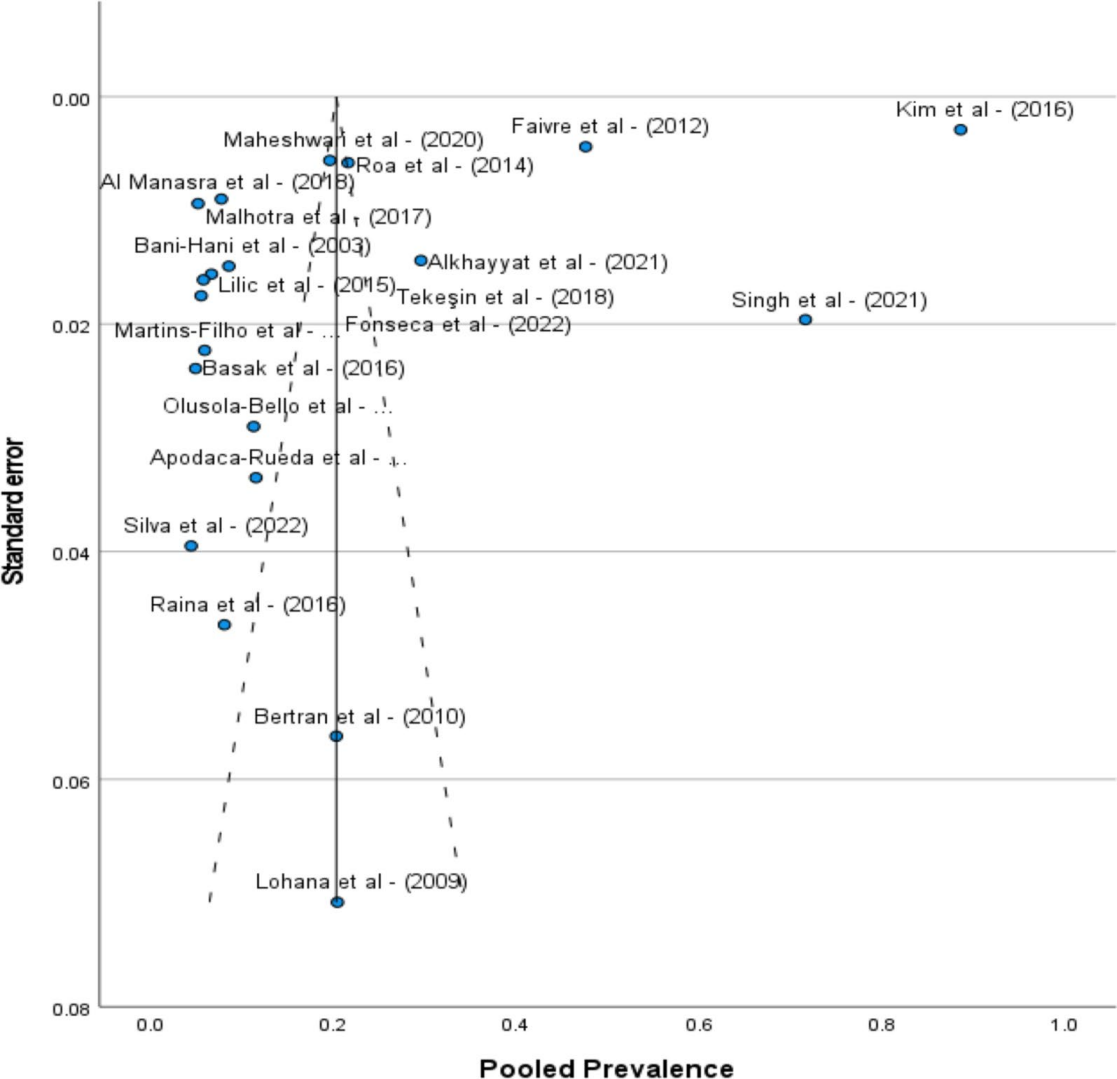
A funnel plot representing distribution of studies. The substantial heterogeneity contributes to the deviation of 3 studies [31–33] and thus the asymmetry in the funnel plot

Notably, the Egger’s regression-based test revealed no publication bias with coefficients for various parameters such as; publication year, geographical region, study duration, study design, sample size, mean age and tumor stage considered. The intercept (Intercept = 2.253) was not statistically significant (*p* = 0.804), suggesting that the asymmetry in the funnel plot may not be indicative of publication bias (Fig. 3). Nonetheless, to account the asymmetry in funnel plot, individual parameters such as publication year, geographical region, study duration, study design, sample size category, mean age category, and tumor stage were analyzed and showed varying levels of significance and influence on the overall effect size. Among the covariates, the sample size indicates a potential influence on the asymmetry in the funnel plot (*p* = 0.040). This suggests that studies with larger sample sizes might be associated with different effect sizes, impacting the overall meta-analysis results. Other covariates, such as study design, geographical region, and tumor stage, also exhibit *p*-values approaching significance as summarized in Table 3.

**Table 3:** Egger’s regression-based analysis

Parameter	Coefficient ± Std Error	t stat	*P* value	95% confidence interval	
				Lower	Upper
(Intercept)	2.25 ± 7.08	0.318	0.804	− 87.653	92.159
Publication year	− 0.003 ± 0.004	− 0.764	0.585	− 0.047	0.042
geographical region	0.16 ± 0.04	4.365	0.143	− 0.302	0.618
Study duration	− 0.11 ± 0.06	− 1.929	0.304	− 0.810	0.596
Study design	− 0.7 ± 0.08	− 9.162	0.069	− 1.670	0.271
Sample size Category	2.94 ± 0.19	15.765	0.040	0.570	5.307
Mean age category	− 0.17 ± 0.03	− 6.360	0.099	− 0.501	0.167
Tumor stage	0.35 ± 0.03	10.940	0.058	− 0.056	0.757

### Subgroup analysis

The subgroup analysis (Table 4) reveals significant variations in the prevalence of gallbladder cancer across regions. The studies in Asia showed a moderately low prevalence [pooled effect = 0.24 ± 0.097 with 95%CI (0.023–0.460), *p* = 0.034]. Meanwhile, in America, the prevalence is slightly higher [pooled effect = 0.14 with 95% CI (0.049–0.236), *p* < 0.05]. Overall, there is a notable trend of varying prevalence across different geographic regions. Further, the studies (*n* = 11) with ≤ 5 years of study duration suggested a notable prevalence (*p* = 0.018). The studies with duration 16–20 years suggest a noteworthy prevalence [pooled effect = 0.25 ± 0.04 with 95% CI (− 0.252–0.762), *p* = 0.099] trend over the specified durations. The results suggest variations in prevalence trends based on study design as significant effects were observed in cross-sectional, descriptive studies (*n* = 7, *p* = 0.013) and retrospective studies (*n* = 10, *p* = 0.057). Studies with small sample sizes (≤ 1000) show substantial prevalence of Gallbladder cancer with a statistically significant effect (*p* = 0.018).

**Table 4:** Subgroup analysis on various variables included in the study

Subgroups	No. of Studies	Pooled effect	t statistics	Sig. (2-tailed)	95% Confidence Interval			
					Lower	Upper	Overall p value	I^2^
Statistics								
*Geographical region*^a^								
Aisa	10	0.24 ± 0.097	2.500	0.034	0.023	0.460	0.03	1.00
America	7	0.14 ± 0.038	3.724	0.010	0.049	0.236	0.01	0.97
*Study duration*^a^								
≤ 5 years	11	0.17 ± 0.059	2.819	0.018	0.035	0.300	0.02	0.99
6–10 years	4	0.17 ± 0.102	1.689	0.190	− 0.152	0.496	0.19	1.00
11–15 years	2	0.47 ± 0.417	1.125	0.463	− 4.829	5.767	0.46	1.00
16–20 years	3	0.25 ± 0.04	6.396	0.099	− 0.252	0.762	0.10	0.96
*Study design*								
Cross-sectional, Descriptive study	7	0.2 ± 0.058	3.518	0.013	0.062	0.347	0.01	1.00
Population-based Cancer Registries	3	0.15 ± 0.044	3.509	0.072	− 0.035	0.340	0.07	0.98
Retrospective study	10	0.22 ± 0.099	2.181	0.057	− 0.008	0.440	0.06	1.00
*Sample size*								
≤ 1000	5	0.12 ± 0.03	3.856	0.018	0.033	0.201	0.02	0.48
1001–5000	9	0.17 ± 0.073	2.272	0.053	− 0.002	0.336	0.05	0.99
≥ 1000	6	0.32 ± 0.129	2.456	0.058	− 0.015	0.650	0.06	1.00
*Mean age*^b^								
≤ 50 years	3	0.05 ± 0.019	2.859	0.104	− 0.027	0.134	0.10	0.00
51–55 year	4	0.3 ± 0.145	2.064	0.131	− 0.162	0.762	0.13	1.00
56–60 year	4	0.13 ± 0.028	4.475	0.021	0.037	0.218	0.02	0.95
61–65 year	2	0.49 ± 0.4	1.215	0.438	− 4.598	5.570	0.44	1.00
66–70 year	3	0.09 ± 0.042	2.147	0.165	− 0.090	0.270	0.16	0.92
≥ 71 years	2	0.18 ± 0.12	1.463	0.382	− 1.352	1.704	0.38	0.99
*Cancer stage*^b^								
II–III	2	0.17 ± 0.123	1.407	0.393	− 1.393	1.739	0.39	0.99
I–IV	6	0.28 ± 0.108	2.578	0.050	0.001	0.555	0.05	1.00
*Risk factors*^b^								
Cholecystitis	3	0.07 ± 0.015	4.527	0.045	0.003	0.135	0.05	0.25
Obesity	2	0.25 ± 0.04	6.396	0.099	− 0.252	0.762	0.10	0.96
Gallstones	8	0.14 ± 0.033	4.138	0.004	0.058	0.212	0.00	0.95
Gallbladder polyps	2	0.08 ± 0.031	2.475	0.244	− 0.313	0.464	0.24	0.70

^a^ Subgroups excluded due to their single study; ^b^ studies from subgroups with missing data were omitted

Notably, the 56–60-year age group shows a significantly higher prevalence [pooled effect = 0.13 ± 0.028 and 95% CI (0.037–0.218) and *p* < 0.05]. Conversely, the 61–65-year and ≥ 71-year groups demonstrate higher pooled effects, but with wider confidence intervals and non-significant results (*p* = 0.165 and *p* = 0.382 respectively). Moreover, the studies covering tumor stages I–IV showed significant results (*p* = 0.05). Lastly, there were varying associations between different risk factors and the prevalence of Gallbladder cancer. Notably, studies on Cholecystitis show a minimal effect (pooled effect = 0.07 ± 0.015 and 95% CI (0.003–0.135), *p* = 0.045) indicating a potential association with lower GBC prevalence. In contrast, obesity exhibits a larger effect size of 0.25 ± 0.04, but with a non-significant *p* = 0.099. Gallstones demonstrate a moderate effect size of 0.14, significantly associated with higher GBC prevalence (*p* = 0.004). Gallbladder polyps, with an effect size of 0.08, show no significant association (*p* = 0.244). Therefore, Gallstones appear to be the risk factor most strongly associated with increased GBC prevalence.

### Heterogeneity analysis

**Fig. 4:**
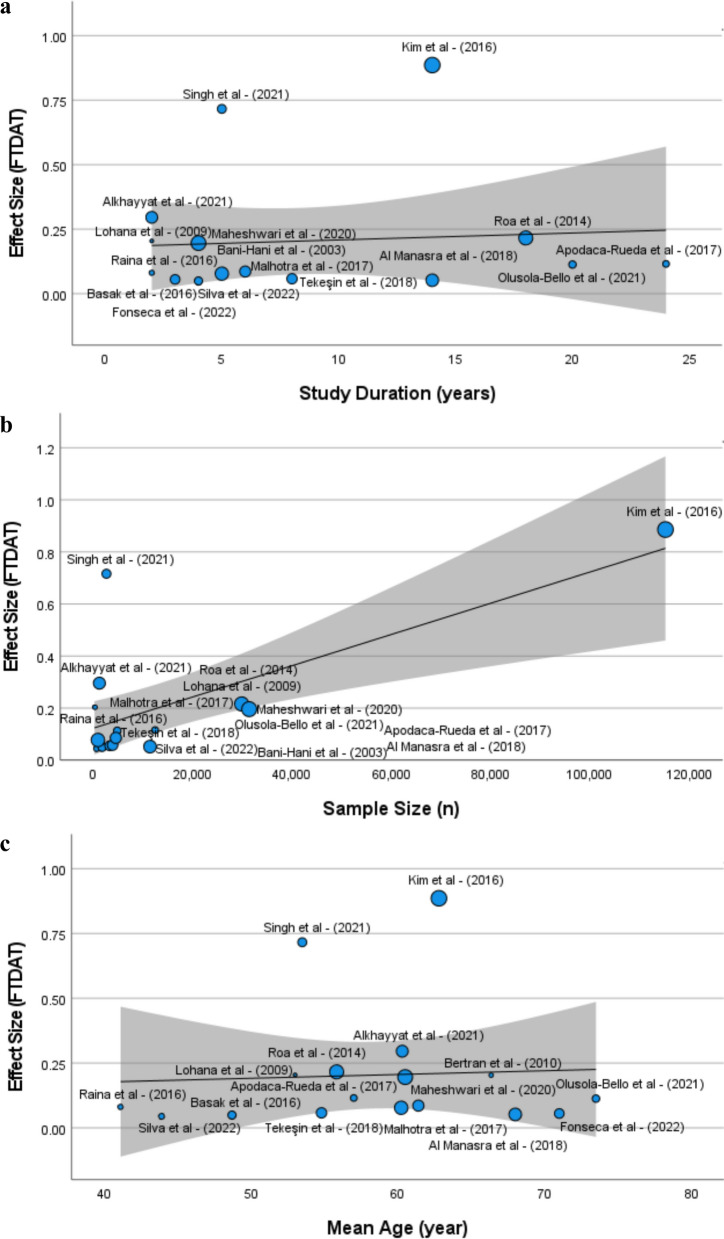
Bubble plots showing the variability of different studies in various moderators

Overall analysis of studies reveals substantial heterogeneity (I^2^ = 0.99) leading to the choice of Random Effect model for current meta-analysis. To assess the potential sources of heterogeneity, we performed meta regression analysis for potential moderators e.g., study duration, sample size, and mean age as shown in Fig. 4a–c.

The analysis indicates significant heterogeneity in the residuals (Q stat = 31.677, *p* = 0.000). The residual heterogeneity (Tau^2^ = 0.024) signifies the amount of true heterogeneity present. There was a high degree of inconsistency among the studies (I^2^ = 98.6%), with proportion of total variability (H^2^ = 72.535) attributed to true heterogeneity. Furthermore, the moderators included in the analysis (study duration, sample size, and mean age) explain a substantial portion of the observed variability (R^2^ = 59.0%). Overall, the results suggest that while these moderators contribute to explaining some heterogeneity, further, variables such as; geographical location, study design and cancer stages contribute to the heterogeneity.

### Quality appraisal

The overall interpretation of the quality appraisal (Supplementary Material: Quality Appraisal) indicates that the majority of studies in our meta-analysis adhered to JBI guidelines, with scores ranging from 6 to 9. Most studies had appropriate sample frames, participant sampling, and statistical analysis, but some exhibited shortcomings in sample, response rate, or description of subjects and settings size [24, 27].

### Correlation analysis

These findings from correlation analysis (Table 5) suggest that the observed prevalence of gallbladder cancer may be influenced by the size of the study population and the duration of the study. It reveals significant associations between prevalence (%) of gallbladder cancer and several other variables. There is a strong positive correlation with sample size (Pearson correlation = 0.750, *p* < 0.01), indicating that studies with larger sample sizes tend to report higher prevalence rates. Additionally, the number of males (Pearson correlation = 0.805, *p* < 0.01) and the number of females (Pearson correlation = 0.806, *p* < 0.01) also show strong positive correlations with prevalence. This suggests that a higher prevalence of gallbladder cancer is associated with studies that include a greater number of male and female participants. On the other hand, there is a weak positive correlation with study duration (Pearson correlation = 0.092, *p* = 0.700), and the correlation with mean age is negligible (Pearson correlation = 0.002, *p* = 0.993). These findings highlight the influence of sample size and gender distribution on the reported prevalence of gallbladder cancer.

**Table 5:** Influence of various variables on prevalence of gallbladder cancer

	Pearson correlation	Sig. (2-tailed)	Covariance	No. of studies
Prevalence (%)	0.98	0.04	254.412	20
Study duration (years)	0.092	0.700	9.645	20
Sample size (n)	0.750	0.000	326,353.993	20
Mean age (year)	0.002	0.993	0.318	18
No. of males	0.805	0.000	136,166.866	17
No. of females	0.806	0.000	90,574.202	17

## Discussion

Our study reveals a significant and robust worldwide prevalence of GBC, with an overall pooled prevalence of 20.3%. The analysis demonstrates considerable variability in prevalence and sample sizes, suggesting a complex landscape for GBC. Subgroup analyses uncover geographical variations, with Asia showing a moderately low prevalence compared to slightly higher rates in America. The influence of study duration, design, sample size, and age on prevalence is explored, with significant findings such as higher prevalence in the 56–60-year age group and significant associations with risk factors like gallstones. Strong positive correlations between prevalence and sample size, as well as gender distribution, are evident, suggesting their impact on reported GBC prevalence.

Our main findings of pooled prevalence of 20.3% contrasts with the global incidence data reported by Sturm et al. [2] and Wi et al. [3], where GBC constitutes approximately 1.3% of total cancer cases and 1.7% of cancer-related deaths worldwide. The apparent disparity arises because the global statistics are population-wide averages, while our meta-analysis focuses specifically on studies investigating high-risk cohorts. Moreover, regional subgroup analysis further supports moderate-to-high prevalence in Asia (pooled effect = 0.24, 95% CI 0.023–0.460, *p* = 0.034) which is consistent with the established higher burden of GBC in certain Asian countries. Similarly, studies focusing on older populations (mean age ≥ 59 years) and advanced cancer stages (I–IV) reported notable prevalence trends. Our findings underscore the influence of targeted population characteristics and risk factors—such as gallstones, which were significantly associated with increased GBC prevalence (*p* = 0.004)—on the observed prevalence rates. Although the global population incidence of GBC remains relatively low, its prevalence among high-risk groups is noted which aligns our findings with the broader epidemiological context.

In our analysis, Kim et al. reported a localized higher prevalence [32] which can be explained by methodological and population-specific factors. Although the study focused on patients undergoing ultrasonography for gallbladder sludge, revealing 14% of cases with tumefactive sludge, which ultimately highlights the selective focus of the study on high-risk individuals.

In contrast, Huang et al. (2020) reported a global age-standardized rate (ASR) of incidence as 2.3 per 100,000 persons in 2018, with the highest rates in Eastern Asia (ASR = 3.0) and the lowest in Middle Africa (0.35). For mortality, the global ASR was 1.7, with the highest rates again in Eastern Asia (ASR = 2.4) and the lowest in Middle Africa (0.29). Huang et al. also highlighted a positive correlation between higher Human Development Index (HDI) and both incidence and mortality rates, with a correlation coefficient (r) of 0.31 and 0.22, respectively [33]. Although counterintuitive at first glance, this relationship may reflect differences in healthcare systems, disease detection rates, and lifestyle risk factors. High-HDI countries often have better healthcare infrastructure, leading to earlier and more frequent diagnoses, which can inflate incidence rates. Additionally, lifestyle factors such as high rates of obesity, smoking, and hypercholesterolemia—also more prevalent in developed nations—contribute to the increased burden of GBC in these regions.

Our results provide a complementary perspective by focusing on prevalence rather than incidence or mortality. The pooled prevalence of GBC in our meta-analysis was 20.3 ± 5.2% (95% CI 9.3–31.3%, *p* = 0.001), with significant geographic variability. Studies from Asia and America demonstrated moderate-to-high prevalence which reflects the influence of regional risk factors such as gallstones and obesity. Our findings align with Huang et al.’s observations regarding the impact of lifestyle and socioeconomic factors on GBC burden.

Interestingly, while our analysis underscores a higher prevalence in populations with predisposing conditions (e.g., gallstones, obesity), the focus on high-risk groups likely skews prevalence estimates upward compared to population-based incidence data reported by Huang et al. Our subgroup analysis revealed that Asian countries demonstrated a moderately low prevalence (pooled effect = 0.24, 95% CI 0.023–0.460, *p* = 0.034), contrasting with the higher incidence rates reported by Huang et al. in Eastern Asia. This discrepancy may arise from differences in study populations, diagnostic criteria, or healthcare access. Furthermore, the association between higher HDI and increased GBC incidence and mortality reflects the interplay of development, lifestyle factors like obesity and metabolic disorders driven by dietary and behavioral changes, and improved diagnostic capabilities, as supported by Huang et al.'s correlations [33] and corroborated by our findings linking obesity and gallstones to higher GBC prevalence.

Notably, a higher prevalence of smoking and overweight correlated with higher GBC rates, with correlation coefficients of 0.26 and 0.20, respectively [34]. The comparison of these results underscores the complexity of GBC epidemiology, with our meta-analysis providing a detailed prevalence perspective and Huang et al. offering insights into incidence, mortality, and their correlations with socioeconomic and lifestyle factors.

As, Huang et al. (2021) reported dynamics of GBC globally observing variations in GBC incidence and mortality worldwide in 2018, with higher rates in more developed countries and among females [33], the association between higher incidence and more developed countries aligns with our meta-analysis, which highlighted geographical variations in GBC prevalence. Notably, their observation of an increasing trend in GBC incidence among specific populations, particularly in males and younger individuals aged < 50 years, provides a temporal perspective that complements our cross-sectional meta-analysis findings. Moreover, findings from Khan et al. (2022) shed light on the characteristics of incidental GBC based on pathological analysis of cholecystectomy specimens. They found a 0.39% incidental GBC rate in 33,467 individuals, with no gender difference. Older age, advanced tumor stages (T2/T3), and factors like cholecystitis and dysplasia were associated with incidental GBC [35]. These results align with our analysis, emphasizing the impact of age and histopathological features on the prevalence and characteristics of gallbladder cancer. In comparison to the study by Phadke et al. (2019), our analyses provide a broader perspective. While Phadke et al. focused on specific regions in India, our analysis captures a global overview, offering a more comprehensive understanding of GBC prevalence. Overall, our study enhances the understanding of GBC prevalence on a global scale, complementing the regional insights provided by Phadke et al. [36].

Jang et al. [37] studied spatial patterns and associated factors of liver and gallbladder cancer incidence in Korea. They identify spatial clusters with high gallbladder cancer incidence rates in the southeastern region. Moreover, Singh et al. focus on 1845 cases, of which 60.97% were diagnosed with GBC. Notably, a majority of GBC cases were from rural backgrounds (62.84%), emphasizing the significance of geographical and lifestyle factors. The mean age of GBC cases was approximately 53.49 years, with a higher prevalence in females (70.37%) than males (29.63%) [31]. The comparison underscores the complementarity of our study, with Singh et al. offering in-depth insights into the characteristics of a specific cohort, while current study contributes to the broader understanding of GBC prevalence trends globally.

Our findings align with Nervi et al.’ [38] study on GBC prevalence in Chile, emphasizing the pivotal role of gallstone disease as a primary risk factor for the disease. Both studies reveal a substantial prevalence of gallstone disease, with Nervi et al. reporting 45% in women and 20% in men older than 20 years in Chile, while our study, encompassing diverse regions, consistently identifies gallstones as a significant risk factor linked to increased gallbladder cancer prevalence. Moreover, the gender-based disparities observed in both studies, with women exhibiting a higher risk, further validate our findings. Moreover, Albert et al. (1985) in a case–control study, provided additional context and reinforcing the significance of gallstones as a major factor in gallbladder cancer risk [39]. The estimated relative risk (RR) reported by Albert et al. supports our findings of gallstones as a crucial risk factor, with their study indicating a substantial RR in both non-Indian RR = 4.4; 95%CI (2.6–7.3) and Indian populations RR = 20.9; 95% CI (8.1–54).

Aldouri et al. [40] add depth to the understanding of risk factors for GBC on gallbladder polyps. Their study reveals that 3.3% of patients had these polyps. They identified risk factors for developing GBC include age above 60 years, presence of gallstones and several others. These results resonate with our analysis, particularly in emphasizing the association between gallstones and GBC. Aldouri et al.’s observation that the prevalence of GBC in patients with GBP is significantly higher among those of Indian ethnic background compared to Caucasians aligning with the notion of ethnic variations in GBC risk highlighted in our study.

Despite the valuable contributions, our study is not without limitations. The retrospective nature of some included studies may introduce biases, and the variability in study durations and designs could contribute to heterogeneity. Furthermore, the correlation analysis provides associations but not causation. Moreover, the lack of data on certain potential influencing factors, such as genetic predispositions and environmental exposures, limits the comprehensive understanding of GBC dynamics. Future studies should consider incorporating analyses of independent risk factors for gallbladder cancer to provide insights into the causal pathways and factors contributing to its onset and mortality.

## Conclusion

The scarcity of GBC epidemiology-specific studies is a notable gap in the existing literature. This meta-analysis represents a pioneering effort to consolidate available data on a cancer type that has been historically overlooked due to its rarity. The significant dearth of dedicated research in this area underscores the need for increased attention to GBC, and our analysis serves as a crucial starting point. By providing a comprehensive overview of prevalence across various regions, study types, and demographic factors, our study opens new avenues for future research in the field of GBC. The identification of regional variations, risk factors, and demographic associations highlights the complexity of this cancer type and emphasizes the importance of targeted investigations.

In conclusion, our meta-analysis contributes significantly to the understanding of gall bladder cancer prevalence. Future research should aim to address the identified limitations by conducting prospective studies with standardized methodologies. Exploring the influence of genetic and environmental factors on prevalence could also provide a more comprehensive picture of gall bladder cancer dynamics.

## Supplementary Information

Additional file 1.

## Data Availability

All data supporting the findings of this study are available within the paper and its supplementary material.
