# Supplementary material for: Epidemiology of gall bladder cancer and its prevalence worldwide: a meta-analysis
Source: Orphanet J Rare Dis. 2025 Mar 27;20:143. doi: 10.1186/s13023-025-03652-0 (PMC11948642; doi:10.1186/s13023-025-03652-0)
Supplement: Supplementary file 1 — Additional file 1. [file 13023_2025_3652_MOESM1_ESM.pdf]

Supplementary Material

Epidemiology of Gall Bladder Cancer and its prevalence worldwide: A Meta-Analysis

Search Strategy

PubMed

("Gallbladder cancer" OR "Gallbladder neoplasms" OR "Cholecystic carcinoma") AND

(Prevalence OR Epidemiology OR Risk factors OR Demographics OR Cancer staging) AND

("Cross-sectional studies" OR "Retrospective studies" OR "Population-based cancer registries" OR "Descriptive studies") AND (Gallstones OR Cholecystitis OR "Gallbladder polyps") AND

(Geographic variation OR "Gender distribution" OR "Age distribution")

Google Scholar

"Gallbladder cancer" OR "Gallbladder neoplasms" OR "Cholecystic carcinoma" AND Prevalence OR Epidemiology OR "Risk factors" OR Demographics OR "Cancer staging" AND "Cross-sectional studies" OR "Retrospective studies" OR "Population-based cancer registries" OR "Descriptive studies" AND Gallstones OR Cholecystitis OR "Gallbladder polyps" AND "Geographic variation" OR "Gender distribution" OR "Age distribution"

Scopus

TITLE-ABS-KEY("Gallbladder cancer" OR "Gallbladder neoplasms" OR "Cholecystic carcinoma") AND TITLE-ABS-KEY(Prevalence OR Epidemiology OR "Risk factors" OR Demographics OR "Cancer staging") AND TITLE-ABS-KEY("Cross-sectional studies" OR "Retrospective studies" OR "Population-based cancer registries" OR "Descriptive studies") AND TITLE-ABS-KEY(Gallstones OR Cholecystitis OR "Gallbladder polyps") AND TITLE-ABS-KEY("Geographic variation" OR "Gender distribution" OR "Age distribution")

Web of Science

TS=("Gallbladder cancer" OR "Gallbladder neoplasms" OR "Cholecystic carcinoma") AND TS(Prevalence OR Epidemiology OR "Risk factors" OR Demographics OR "Cancer staging") AND TS("Cross-sectional studies" OR "Retrospective studies" OR "Population-based cancer registries" OR "Descriptive studies") AND TS(Gallstones OR Cholecystitis OR "Gallbladder polyps") AND TS("Geographic variation" OR "Gender distribution" OR "Age distribution")

Quality Appraisal

JBI Checklist

Q1. Was the sample frame appropriate to address the target population?
Q2. Were study participants sampled in an appropriate way?
Q3. Was the sample size adequate?
Q4. Were the study subjects and the setting described in detail?
Q5. Was the data analysis conducted with sufficient coverage of the identified sample?
Q6. Were valid methods used for the identification of the condition?
Q7. Was the condition measured in a standard, reliable way for all participants?
Q8. Was there appropriate statistical analysis?
Q9. Was the response rate adequate, and if not, was the low response rate managed appropriately?

**Study**	**Q1**	**Q2**	**Q3**	**Q4**	**Q5**	**Q6**	**Q7**	**Q8**	**Q9**	**Assessment score**
Silva et al	✓	✓	✗	✓	✓	✓	✓	✓	✓	08
Basak et al	✓	✓	✓	✓	✓	✓	✓	✓	✗	08
Al Manasra et al	✓	✗	✗	✓	✓	✓	✓	✓	✓	07
Fonseca et al	✓	✓	✓	✓	✓	✓	✓	✓	✓	09
Tekeşin et al	✓	✓	✓	✓	✓	✓	✓	✓	✓	09
Martins-Filho et al	✓	✗	✓	✗	✓	✓	✓	✓	✓	07
Lilic et al	✓	✓	✓	✓	✓	✓	✗	✓	✓	08
Raina et al	✓	✓	✗	✓	✓	✓	✓	✓	✓	08
Malhotra et al	✓	✓	✓	✓	✓	✓	✓	✓	✓	09
Bani-Hani et al	✓	✓	✓	✓	✓	✓	✓	✓	✓	09
Olusola-Bello et al	✓	✓	✓	✓	✓	✓	✓	✓	✗	08
Apodaca-Rueda et al	✓	✓	✗	✓	✓	✗	✓	✓	✗	06

Maheshwari et al	✗	✓	✓	✓	✓	✓	✓	✓	✓	08
Lohana et al	✓	✓	✗	✓	✓	✓	✓	✓	✓	08
Bertran et al	✓	✓	✗	✓	✓	✓	✓	✗	✗	06
Roa et al	✓	✓	✓	✓	✓	✓	✓	✓	✓	09
Alkhayyat et al	✓	✓	✓	✓	✓	✓	✓	✓	✓	09
Faivre et al	✓	✓	✓	✓	✓	✓	✓	✓	✓	09
Singh et al	✓	✓	✓	✓	✓	✓	✓	✓	✓	09
Kim et al	✓	✓	✓	✓	✓	✓	✓	✓	✓	09

Data Extracted

Sr.	Study ID	Country/Location	Time Period	Sample Size	Affected	Median Age	Mean Age Category	Mean Age	Risk Factors	Cancer Stage	Prevalence (%)
18	Kim et al - (2016)	Korea	2001 - 2015	115178	69107	N/A	3	62.8	N/A	0	60
8	Basak et al - (2016)	Turkey	2009 - 2013	1747	4	48.7	0	48.7	Cholecystitis and Gallstones	1	0.23
2	Alkhayyat et al - (2021)	Unites States	1999 - 2019	4790	407	60	5	73.5	gallstones, Obesity and diabetes mellitus	1	8.5
11	Olusola-Bello et al - (2021)	Nigeria	2015 - 2017	1191	15	N/A	2	60.3	Gall Stones	2	1.25
12	Tekeşin et al - (2018)	Turkey	2009 - 2017	3856	13	58	1	54.8	N/A	3	0.33
13	Singh et al - (2021)	India	2009 - 2014	2610	1125	N/A	1	53.49	N/A	4	43.1
1	Apodaca-Rueda et al - (2017)	Brazil	2010 - 2015	893	12	59	2	60.23	Gall Stones, Gallbladder polyps	4	1.3
6	Bani-Hani et al - (2003)	Jordan	1994 - 2000	4502	33	N/A	3	61.4	Cholecystitis, Gallstones	4	0.73
3	Bertran et al - (2010)	Chile	1998 - 2002	317	13	54.5	4	66.36	gallbladder stone	4	4
7	Lilic et al - (2015)	New Zealand	2003 - 2013	4128	18	N/A	4	70.6	Adenocarcinoma	4	0.44
16	Faivre et al - (2012)	Europe	1995 - 2002	50646	10636	N/A		N/A	N/A	4	21
14	Raina et al - (2016)	India	2014 - 2016	464	3	N/A	0	41.1	N/A		0.6
19	Silva et al - (2022)	Brazil	2018 - 2022	642	103	N/A	0	43.9	Cholecystitis		0.16
17	Lohana et al - (2009)	Pakistan	2006 - 2008	200	8	N/A	1	53	Gall Stones		4
4	Roa et al - (2014)	Chile	1987 - 2005	29840	1373	56.5	1	55.83	cholesterolosis,obesity		4.6
10	Maheshwari et al - (2020)	India	2014 - 2018	31355	1191	60	2	60.5	N/A		3.8
9	Malhotra et al - (2017)	India	1988 - 2012	12410	74	60.13	2	57	N/A		0.6

5	Al Manasra et al - (2018)	Jordan	2002 - 2016	11391	31	N/A	4	68	Gallstones and Gallbladder polyps		0.27
20	Fonseca et al - (2022)	Chile	2016 - 2019	3270	10	N/A	5	71	N/A		0.3
15	Martins-Filho et al - (2015)	Brazil	2007 - 2010	2018	7	N/A		N/A	N/A		0.34

PRISMA 2020 for Abstracts Checklist

Section and Topic	Item #	Checklist item	Reported (Yes/No)
**TITLE**			
Title	1	Identify the report as a systematic review.	Pg#1; Line#2-3
**BACKGROUND**			
Objectives	2	Provide an explicit statement of the main objective(s) or question(s) the review addresses.	Pg#2; Line#36-37
**METHODS**			
Eligibility criteria	3	Specify the inclusion and exclusion criteria for the review.	Pg#2; Line#38-39
Information sources	4	Specify the information sources (e.g. databases, registers) used to identify studies and the date when each was last searched.	Pg#2; Line#38
Risk of bias	5	Specify the methods used to assess risk of bias in the included studies.	Pg#2; Line#39-41
Synthesis of results	6	Specify the methods used to present and synthesise results.	Pg#2; Line#37-38
**RESULTS**			
Included studies	7	Give the total number of included studies and participants and summarise relevant characteristics of studies.	Pg#2; Line#41
Synthesis of results	8	Present results for main outcomes, preferably indicating the number of included studies and participants for each. If meta-analysis was done, report the summary estimate and confidence/credible interval. If comparing groups, indicate the direction of the effect (i.e. which group is favoured).	Pg#2; Line#43-49
**DISCUSSION**			
Limitations of evidence	9	Provide a brief summary of the limitations of the evidence included in the review (e.g. study risk of bias, inconsistency and imprecision).	Pg#2; Line#49
Interpretation	10	Provide a general interpretation of the results and important implications.	Pg#2; Line#50-53
**OTHER**			
Funding	11	Specify the primary source of funding for the review.	Not applicable
Registration	12	Provide the register name and registration number.	Not applicable

From: Page MJ, McKenzie JE, Bossuyt PM, Boutron I, Hoffmann TC, Mulrow CD, et al. The PRISMA 2020 statement: an updated guideline for reporting systematic reviews. BMJ 2021;372:n71. doi: 10.1136/bmj.n71

For more information, visit: http://www.prisma-statement.org/

PRISMA 2020 Checklist

Section and Topic	Item #	Checklist item	Location where item is reported
**TITLE**			
Title	1	Identify the report as a systematic review.	Pg#1; Line#2-3
**ABSTRACT**			
Abstract	2	See the PRISMA 2020 for Abstracts checklist.	Added as **Suppl. Mater.**
**INTRODUCTION**			
Rationale	3	Describe the rationale for the review in the context of existing knowledge.	Pg#4; Line#77-79, 84-88
Objectives	4	Provide an explicit statement of the objective(s) or question(s) the review addresses.	Pg#5; Line#89-92
**METHODS**			
Eligibility criteria	5	Specify the inclusion and exclusion criteria for the review and how studies were grouped for the syntheses.	Pg#5; Line#102-109
Information sources	6	Specify all databases, registers, websites, organisations, reference lists and other sources searched or consulted to identify studies. Specify the date when each source was last searched or consulted.	Pg#5; Line#96-100
Search strategy	7	Present the full search strategies for all databases, registers and websites, including any filters and limits used.	Added as **Suppl. Mater.**
Selection process	8	Specify the methods used to decide whether a study met the inclusion criteria of the review, including how many reviewers screened each record and each report retrieved, whether they worked independently, and if applicable, details of automation tools used in the process.	Pg#5; Line#102
Data collection process	9	Specify the methods used to collect data from reports, including how many reviewers collected data from each report, whether they worked independently, any processes for obtaining or confirming data from study investigators, and if applicable, details of automation tools used in the process.	Pg#6; Line#111
Data items	10a	List and define all outcomes for which data were sought. Specify whether all results that were compatible with each outcome domain in each study were sought (e.g. for all measures, time points, analyses), and if not, the methods used to decide which results to collect.	Pg#6; Line#111-114
	10b	List and define all other variables for which data were sought (e.g. participant and intervention characteristics, funding sources). Describe any assumptions made about any missing or unclear information.	Not applicable
Study risk of bias assessment	11	Specify the methods used to assess risk of bias in the included studies, including details of the tool(s) used, how many reviewers assessed each study and whether they worked independently, and if applicable, details of automation tools used in the process.	Pg#7; Line#131-135
Effect measures	12	Specify for each outcome the effect measure(s) (e.g. risk ratio, mean difference) used in the synthesis or presentation of results.	Pg#7; Line#124-130
Synthesis methods	13a	Describe the processes used to decide which studies were eligible for each synthesis (e.g. tabulating the study intervention characteristics and comparing against the planned groups for each synthesis (item #5)).	Pg#5; Line#102-109
	13b	Describe any methods required to prepare the data for presentation or synthesis, such as handling of missing summary statistics, or data conversions.	Pg#7; Line#124-130
	13c	Describe any methods used to tabulate or visually display results of individual studies and syntheses.	Pg#7; Line#135-136
	13d	Describe any methods used to synthesize results and provide a rationale for the choice(s). If meta-analysis was performed, describe the model(s), method(s) to identify the presence and extent of statistical heterogeneity, and software package(s) used.	Pg#6; Line#123, Pg#7; Line#131-135
	13e	Describe any methods used to explore possible causes of heterogeneity among study results (e.g. subgroup analysis, meta-regression).	Pg#7; Line#131-135
	13f	Describe any sensitivity analyses conducted to assess robustness of the synthesized results.	Pg#6; Line#121-122

PRISMA 2020 Checklist

Section and Topic	Item #	Checklist item	Location where item is reported
Reporting bias assessment	14	Describe any methods used to assess risk of bias due to missing results in a synthesis (arising from reporting biases).	Pg#7; Line#134-136
Certainty assessment	15	Describe any methods used to assess certainty (or confidence) in the body of evidence for an outcome.	Pg#6; Line#129-131
**RESULTS**			
Study selection	16a	Describe the results of the search and selection process, from the number of records identified in the search to the number of studies included in the review, ideally using a flow diagram.	Pg#7; Line#140-141, **Figure 1**, **Table 2**.
	16b	Cite studies that might appear to meet the inclusion criteria, but which were excluded, and explain why they were excluded.	**Table 2**
Study characteristics	17	Cite each included study and present its characteristics.	Pg#7; Line#142-150, **Table 2** & **Table 1**
Risk of bias in studies	18	Present assessments of risk of bias for each included study.	Pg#11; Line#172-185
Results of individual studies	19	For all outcomes, present, for each study: (a) summary statistics for each group (where appropriate) and (b) an effect estimates and its precision (e.g. confidence/credible interval), ideally using structured tables or plots.	**Table 4**
Results of syntheses	20a	For each synthesis, briefly summarise the characteristics and risk of bias among contributing studies.	**Table 3**
	20b	Present results of all statistical syntheses conducted. If meta-analysis was done, present for each the summary estimate and its precision (e.g. confidence/credible interval) and measures of statistical heterogeneity. If comparing groups, describe the direction of the effect.	**Table 4**
	20c	Present results of all investigations of possible causes of heterogeneity among study results.	**Figure 4**
	20d	Present results of all sensitivity analyses conducted to assess the robustness of the synthesized results.	**Table 5**
Reporting biases	21	Present assessments of risk of bias due to missing results (arising from reporting biases) for each synthesis assessed.	Pg#15; Line#224-236
Certainty of evidence	22	Present assessments of certainty (or confidence) in the body of evidence for each outcome assessed.	**Table 4**
**DISCUSSION**			
Discussion	23a	Provide a general interpretation of the results in the context of other evidence.	Pg#20-22; Line#268-324
	23b	Discuss any limitations of the evidence included in the review.	Pg#22; Line#325-330
	23c	Discuss any limitations of the review processes used.	Pg#22; Line#325-330
	23d	Discuss implications of the results for practice, policy, and future research.	Pg#23; Line#341-345
**OTHER INFORMATION**			
Registration and protocol	24a	Provide registration information for the review, including register name and registration number, or state that the review was not registered.	Not applicable
	24b	Indicate where the review protocol can be accessed, or state that a protocol was not prepared.	Not applicable
	24c	Describe and explain any amendments to information provided at registration or in the protocol.	Not applicable
Support	25	Describe sources of financial or non-financial support for the review, and the role of the funders or sponsors in the review.	Not applicable
Competing interests	26	Declare any competing interests of review authors.	Authors declare no competing interests.

PRISMA 2020 Checklist

Section and Topic	Item #	Checklist item	Location where item is reported
Availability of data, code and other materials	27	Report which of the following are publicly available and where they can be found: template data collection forms; data extracted from included studies; data used for all analyses; analytic code; any other materials used in the review.	All the data synthesized or used is added as supplementary material.

From: Page MJ, McKenzie JE, Bossuyt PM, Boutron I, Hoffmann TC, Mulrow CD, et al. The PRISMA 2020 statement: an updated guideline for reporting systematic reviews. BMJ 2021;372:n71. doi: 10.1136/bmj.n71

For more information, visit: http://www.prisma-statement.org/
